# Synthetic MIR143-3p Suppresses Cell Growth in Rhabdomyosarcoma Cells by Interrupting RAS Pathways Including PAX3–FOXO1

**DOI:** 10.3390/cancers12113312

**Published:** 2020-11-10

**Authors:** Nobuhiko Sugito, Kazuki Heishima, Yuko Ito, Yukihiro Akao

**Affiliations:** 1 United Graduate School of Drug Discovery and Medical Information Sciences, Gifu University, 1-1 Yanagido, Gifu 501-1194, Japan; nsugito@gifu-u.ac.jp (N.S.); heishima@gifu-u.ac.jp (K.H.); 2 Department of Anatomy and Cell Biology, Division of Life Sciences, Osaka Medical College, 2-7 Daigaku-machi, Takatsuki, Osaka 569-8686, Japan; an1006@osaka-med.ac.jp

**Keywords:** RMS, PAX3-FOXO1, MIR143-3p, KRAS, NRAS

## Abstract

**Simple Summary:**

Rhabdomyosarcoma (RMS) is a soft tissue sarcoma with embryonal (ERMS) and alveoral (ARMS) features, most frequently found in children. ARMS has the worse prognosis due to the formation of the chimeric *PAX3–FOXO1* gene. New therapies are needed for the treatment of ARMS. The aim of this study is to evaluate the anticancer effect of chemically-modified MIR143-3p#12 (CM-MIR143#12) on RMS. The ectopic expression of CM-MIR143#12 induced a cell growth suppression by silencing not only *KRAS*, *AKT*, and *ERK* but also the *PAX3–FOXO1* chimeric gene, and KRAS networks could control the expression of chimeric *PAX3–FOXO1* in ARMS cells. Moreover, CM-MIR143#12 also silenced NRAS mutant in ERMS RD cells. CM-MIR143#12 can be a new nucleic acid medicine for the treatment of RMS by impairing the RAS networks including PAX3–FOXO1.

**Abstract:**

Rhabdomyosarcoma (RMS) is a soft tissue sarcoma most frequently found in children. In RMS, there are two major subtypes, embryonal RMS (ERMS) and alveolar RMS (ARMS). ARMS has the worse prognosis of the two owing to the formation of the chimeric *PAX3–FOXO1* gene. A novel therapeutic method is required for treating ARMS. In our previous study, we found that the ectopic expression of chemically modified MIR143-3p#12 (CM-MIR143#12), which is RNase-resistant and shows the highest anti-proliferation activity among the synthesized MIR143 derivatives that were tested, induces significant cell growth suppression by targeting *KRAS*, *AKT*, and *ERK* in colorectal cancer cells. The expression of MIR143-3p in RMS was dramatically downregulated compared with that of normal tissue. Ectopic expression of CM-MIR143#12 in RMS cells resulted in a significant growth inhibitory effect through the induction of apoptosis and autophagy. Interestingly, we found that CM-MIR143#12 also silenced the expression of chimeric *PAX3–FOXO1* directly and, using siR-KRAS or siR-AKT, that KRAS networks regulated the expression of PAX3–FOXO1 in ARMS cells. In ERMS harboring NRAS mutation, CM-MIR143#12 silenced mutated *NRAS*. These findings indicate that CM-MIR143#12 efficiently perturbed the RAS signaling pathway, including the ARMS-specific KRAS/PAX3–FOXO1 networks.

## 1. Introduction

Rhabdomyosarcoma (RMS) is a soft tissue malignant tumor that develops with the highest frequency in childhood. RMS is classified into two types: embryonal RMS (ERMS) and alveolar RMS (ARMS) [1]. ARMS has a poor prognosis [2] because ARMS cells harbor chromosomal translocation t(2;13)(q35;q14) or t(1;13)(q35;q14), which results in the formation of the characteristic chimeric gene through the fusion of *PAX3* or *PAX7* with *FOXO1* that is involved in the pathogenesis of ARMS [3,4,5,6]. Approximately 60% of ARMS cases are PAX3–FOXO1-positive and 20% are PAX7–FOXO1-positive [7]. Moreover, it was reported that 40% of clinical ERMS samples have mutations in genes of the FGFR4/RAS pathway [8,9]. Additionally, it was reported that mutations of RAS were found in 22.4% of fusion-negative RMS cases (NRAS, 11.7%; KRAS, 6.4%; HRAS, 4.3%) [8]. RMS cell lines harboring RAS mutation were strongly dependent on the RAS/RAF/MEK pathway. On the other hand, the phenotype of non-mutated RAS in RMS cells depends on other pathways such as PAX3–FOXO1/FGFR4 [10] and PI3K/AKT/mTOR [11]. A novel therapeutic method that systemically inactivates these pathways is thus required for the treatment of RMS [12,13].

MicroRNAs (MIRNAs; MIRs) are a class of small non-coding RNAs that regulate the expression of genes by binding to mRNAs and inhibiting their translation [14,15]. Moreover, there are many reports documenting the roles of MIRNAs in the pathogenesis of cancer [16,17]. In RMS, the most commonly reported MIRNA is MIR206. MIR206 is a muscle-tissue-specific MIRNA that is available as a biomarker of RMS [18] and is involved in muscle differentiation [19,20]. MIR1, which is in the same family as MIR206, showed anticancer effects by targeting *PTBP1* and regulating energy metabolism in RMS [21]. MIRNA has the potential to target genes that cannot be targeted by conventional molecularly targeted drugs. Therefore, MIRNA may be used as a new form of therapeutic drug directed toward cancers lacking effective treatment approaches. So far, the focus has been on the development of RNA medicines, i.e., the replacement of tumor suppressor (TS)-MIRNAs that target plural genes involved in growth signaling pathways [22,23,24]. Among these TS-MIRNAs, MIR143 is a typical representative whose poor expression is associated with a variety of cancers [25,26,27,28,29]. MIR143 is a potential therapeutic drug for RMS because 15% of RMS patients have a mutation in their *TP53* gene [9], which encodes one of the transcription factors of the MIR143/145 cluster at chromosome 5q33 [30], resulting in the downregulation of MIR143 expression. Furthermore, MIR143-3p induces apoptosis [31,32] and inhibits proliferation, migration, and invasion in osteosarcoma cells [33,34]. We recently reported that the ectopic expression of the chemically modified MIR143-3p#12 (CM-MIR143#12) induces significant inhibition of cancer cell growth through the targeting of *KRAS*, *AKT*, and *ERK* in colorectal [35], bladder [36], and gastric cancer cells [37]. CM-MIR143#12 was developed from among more than 100 kinds of chemically modified MIR143-3p derivatives. Only the guide strand of wild type MIR143 it was c modified using various chemical modifications, such as 2′-fluorine, 2′-methoxy group, phosphorylation, and phosphorothioate, were used (Figure S1A). Moreover, it is strikingly stable in serum (Figure S1B). CM-MIR143#12 exerts anticancer activity with an IC50 of 1.3 nM in KRAS-mutated DLD-1 cells. Our findings clearly show that the on-target effects of CM-MIR143#12 were manifested by interfering with the expression of *KRAS* and key genes in KRAS networks such as *AKT*, *ERK1/2*, and *SOS1*. By using CM-MIR143#12, we were able to clearly show the involvement of target genes related to KRAS networks including *SOS1* of the KRAS-activating system, and KRAS-positive circuit, which is a recruitment system of *KRAS* mRNA from PI3K/AKT and MAPK signaling pathways [35].

In this present study, we found that CM-MIR143#12 also silenced the *PAX3–FOXO1* chimeric gene, and that KRAS networks could control the expression of chimeric *PAX3–FOXO1* in ARMS cells. In addition, CM-MIR143#12 efficiently silenced NRAS mutant in ERMS RD cells. We demonstrated the possibility that CM-MIR143#12 can be a new nucleic acid medicine for the treatment of RMS by efficiently impairing the KRAS networks/PAX3–FOXO1 cascade.

## 2. Results

### 2.1. Downregulated Expression of MIR143-3p in RMS Cell Lines

**Figure 1:**
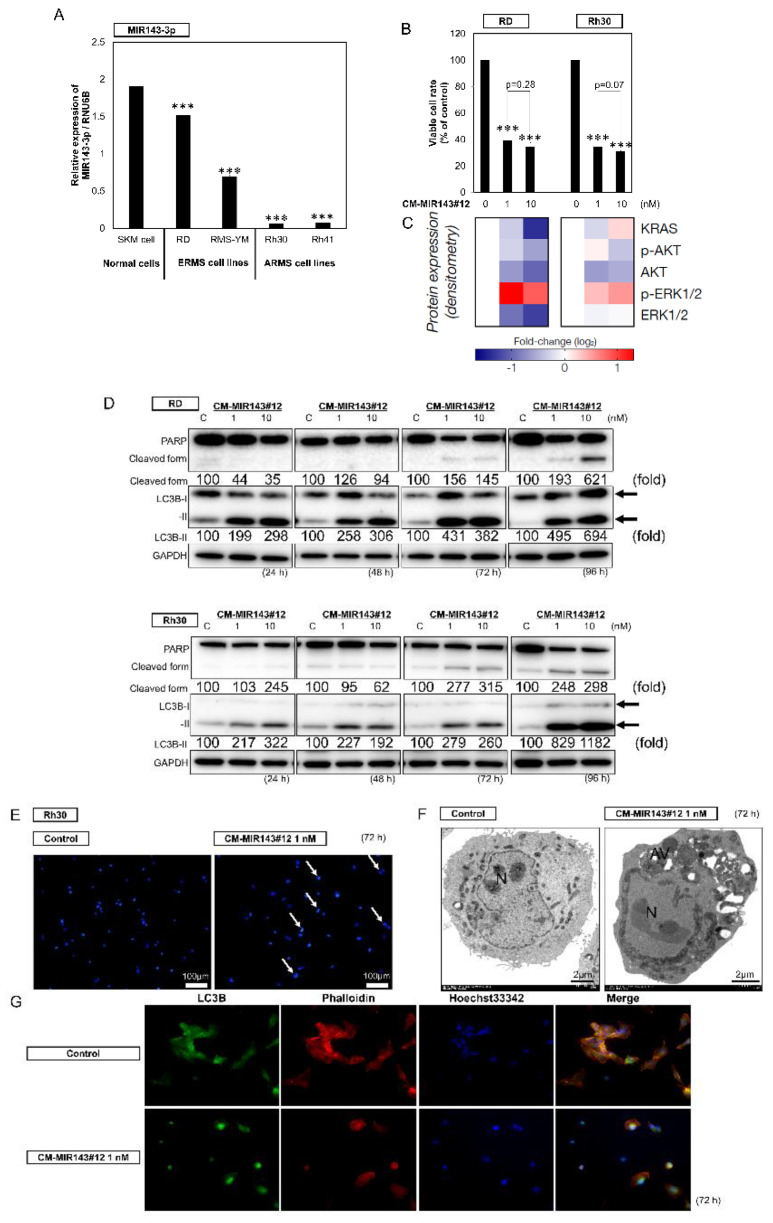
(**A**) Relative expression levels of MIR143 in normal skeletal muscle (SKM) cells and rhabdomyosarcoma cell lines (RD, Rhabdomyosarcoma (RMS)-YM, Rh30, and Rh41). (**B**–**D**) Effects of ectopic expression of CM-MIR143#12 on viability of RMS cells (**B**; Figure S2C) and expression of KRAS, AKT, ERK1/2 (**C**; Figure S2D), and levels of PARP, as an apoptotic marker, and LC3B, as an autophagic marker, as estimated using Western blot analysis (**D**) at 24, 48, 72, and 96 h after transfection of RD and Rh30 cells with CM-MIR143#12 at a concentration of 1 or 10 nM. (**E**) Hoechst 33,342 staining of Rh30 cells at 72 h after transfection with control RNA (1 nM; left) or CM-MIR143#12 (1 nM; right). Typical apoptotic features, such as chromatin condensation and fragmentation of nuclei, were observed (white arrows). (**F**) Morphological examination using electron microscopy. Rh30 cells were treated with control RNA or CM-MIR143 (1 nM) for 72 h. N: fragmented nuclei; AV: autophagosome. (**G**) Immunofluorescence of LC3B at 72 h after transfection of Rh30 cells with CM-MIR143#12 (1 nM). LC3B: green; cytoskeleton (phalloidin): red; nucleus (Hoechst 33342): blue. Results are presented as the mean ± SD; *** *p* < 0.001.

We first evaluated the expression levels of MIR143-3p in RMS cell lines (RD, RMS-YM, Rh30, and Rh41) compared with those in normal skeletal muscle (SKM) cells. The expression of MIR143-3p was significantly downregulated in all RMS cell lines compared with that in normal SKM cells (Figure 1A).

### 2.2. Growth Inhibitory Effect of CM-MIR143#12 on RMS Cells

To clarify the functional roles of MIR143-3p in RMS, we next evaluated the growth inhibitory effect of MIR143-3p on the RMS cell lines by using a chemically modified MIR143-3p#12 (CM-MIR143#12; Figure S1A), which has a comparatively better affinity for its target genes and better RNase tolerance (Figure S1B). In order to examine the effect of CM-MIR143#12 on the growth of RMS cell lines, we transfected RD, RMS-YM, Rh30, and Rh41 cells with CM-MIR143#12. As shown in Figure S2A, the growth of all RMS cell lines was significantly inhibited at 1 nM CM-MIR143#12 for 72 h. Moreover, CM-MIR143#12 did not affect the growth of normal fibroblast cells (Figure S2B). We then chose RD (ERMS) and Rh30 (ARMS) cell lines to compare its effect on each type of RMS because they represent the two main RMS subtypes which have point mutations at *TP53* [38], and because they are the two cell lines that showed the highest sensitivity to CM-MIR143#12 among the analyzed RMS cell lines (Figure S2A). Transfection of either cell line with CM-MIR143#12 induced a significant decrease in the number of viable cells compared with that of the control RNA (Figure 1B and Figure S1SC). Of note, the minimal tested concentration (1 nM) of CM-MIR143#12 was sufficient to induce significant cytotoxicity in both RMS cell lines. To confirm whether CM-MIR143#12 decreased the protein expression levels of KRAS, AKT, and ERK1/2, whose genes are known to be possible targets of MIR143 [35], we examined the protein abundance using Western blot analysis. Expectedly, RD and Rh30 cells transfected with CM-MIR143#12 showed decreased expression levels of KRAS, AKT, and ERK1/2 (Figure 1C and Figure S1D). Furthermore, the RMS cells transfected with CM-MIR143#12 displayed phenotypes of both apoptotic and autophagy, which was also shown by Western blot analysis, which revealed that the transfection with CM-MIR143#12 increased the cleaved form of PARP and the transition of LC3B-I to LC3B-II (Figure 1D). Hoechst 33,342 staining revealed these cells also had morphological changes indicative of apoptosis, such as condensed chromatin and nuclear fragmentation (Figure 1E). Electron microscopy verified the apoptotic changes in the nuclei; interestingly, these cells simultaneously showed autophagic features, i.e., the presence of autophagosomes in the same cell (Figure 1F). Consistent with these morphological changes, immunocytochemistry revealed increased expression of LC3B in certain apoptotic CM-MIR143#12-transfected cells (Figure 1G). Thus, exogenous CM-MIR143#12 likely induced combined autophagy/apoptosis cell death.

### 2.3. AKT Inhibitor Induced Apoptosis and Autophagy in RMS Cells

**Figure 2:**
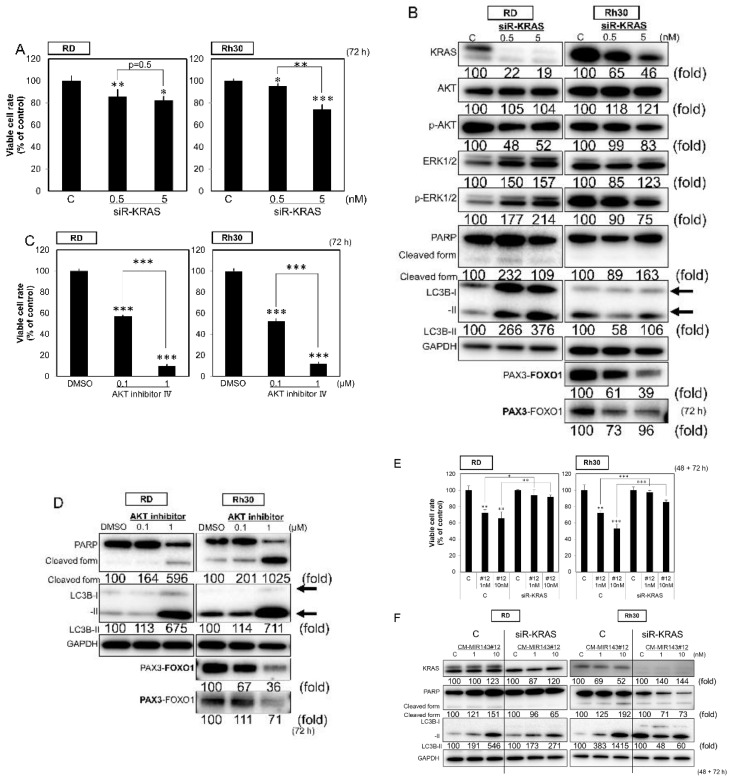
(**A**,**B**) Effects of ectopic expression of siR-KRAS on viability of RMS cells (**A**) and expression of KRAS, AKT, ERK1/2, PARP, LC3B, and PAX3–FOXO1 estimated by Western blot analysis (**B**) at 72 h after treatment of RD and Rh30 cells with siR-KRAS at a concentration of 0.5 or 5 nM. (**C**,**D**) Effects of AKT inhibitor IV on the viability of RMS cells (**C**) and the expression of PARP, LC3B, and PAX3–FOXO1 as estimated by Western blot analysis (**D**) at 72 h after treatment of RD and Rh30 cells with AKT inhibitor IV at a concentration of 0.1 or 1 µM. (**E**,**F**) Effects of ectopic expression of CM-MIR143#12 on the viability of KRAS-knockdown RMS cells (**E**) and expression of KRAS, PARP, and LC3B estimated by Western blot analysis (**F**) in RD and Rh30 cells treated with siR-KRAS (1 nM) for 48 h followed by CM-MIR143#12 transfection at a concentration of 1 or 10 nM for 72 h. PAX3–FOXO1 was detected using the antibody indicated in bold. Results are presented as the mean ± SD; * *p* < 0.05; ** *p* < 0.01; *** *p* < 0.001.

We examined the association between cell death and the expression of target genes downregulated by CM-MIR143#12. As a result, the knockdown of KRAS using siR-KRAS slightly inhibited cell growth in both RD and Rh30 cell lines (Figure 2A); however, siR-KRAS did not induce apparent apoptosis in either cell line (Figure 2B). On the other hand, inhibition of the AKT pathway using AKT inhibitor IV significantly suppressed cell growth with apoptosis and autophagy in both cell lines, even at a 1 µM (Figure 2C,D). In addition to AKT inhibitor IV, the knockdown of AKT using siR-AKT showed similar effects, although weaker than the inhibitor (Figure S3). Moreover, the inhibition of cell proliferation and the induction of apoptosis by CM-MIR143#12 was abolished in siR-KRAS-knockdown RD and Rh30 cells (Figure 2E,F). Importantly, siR-KRAS and AKT inhibitor IV induced the downregulation of PAX3–FOXO1 in Rh30 cells (Figure 2B,D). These findings suggest that CM-MIR143#12 induces apoptosis and autophagy by inhibiting the PI3K/AKT pathway, the downstream signal pathway of KRAS.

### 2.4. CM-MIR-143#12 Inhibits Cell Growth by Silencing NRAS in NRAS-Mutated RD Cell Lines

**Figure 3:**
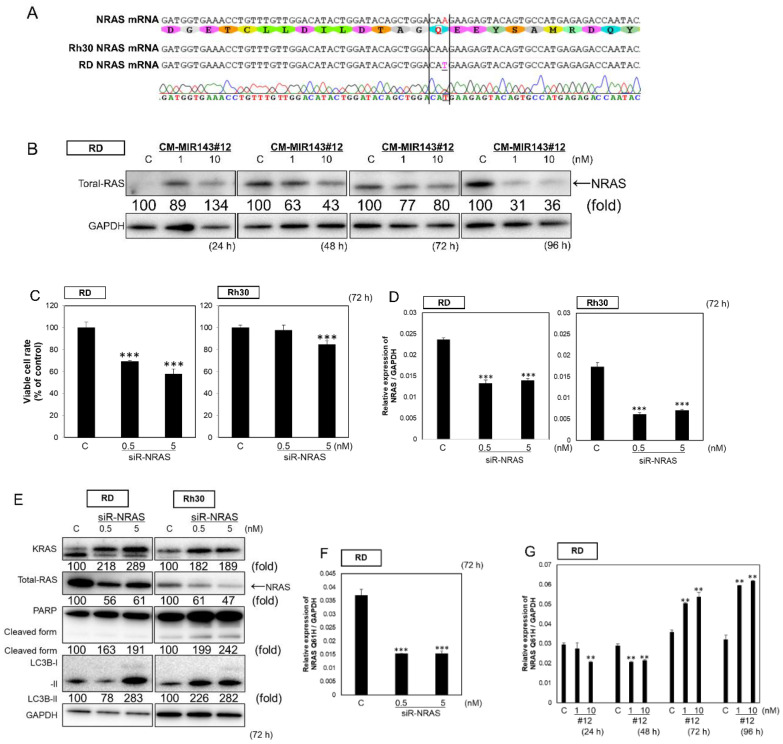
(**A**) Sequence verification of *NRAS* in RD and Rh30 cells. (**B**) Time course of protein expression of NRAS in RD cells at 24, 48, 72, and 96 h after transfection with CM-MIR143#12 (1, 10 nM). (**C**,**D**,**E**) Effects of ectopic expression of siR-NRAS on the cell viability of RMS cells (**C**), the mRNA expression of total *NRAS* estimated by qPCR analysis (**D**), and the protein expression of KRAS, NRAS, PARP, and LC3B estimated by Western blot analysis 72 h after treatment of RD (**E**) and RD cells with siR-NRAS at a concentration of 0.5 or 5 nM (**F**,**G**), changes in *NRAS*-Q61H mRNA levels in siR-NRAS (**F**) or CM-MIR143#12 (**G**)-transfected RD cells. Results are presented as the mean ± SD; ** *p* < 0.01; *** *p* < 0.001.

The RD cell line harbors an NRAS-Q61H mutation (Figure 3A). Of note, in the nucleic acid sequencing of RD cells, the A and T peaks of that point mutation appear to overlap (Figure 3A). Therefore, we examined the steady-state mRNA expression levels of wild type *NRAS* and *NRAS*-Q61H in RD and Rh30 cells by qPCR. *NRAS* was heterogeneously expressed with the expression of both wild type *NRAS* and *NRAS*-Q61H observed in RD cells (Figure S4). Moreover, MIR143-3p targets *NRAS* [39]. Then, we verified whether CM-MIR143#12 inhibited the expression of NRAS in RD cells. As expected, the expression of NRAS was downregulated 48 h after the transfection of RD cells with CM-MIR143#12 (Figure 3B). Next, we performed *NRAS* knockdown to test the effects of NRAS in RD and Rh30 cells. siR-NRAS inhibited cell growth at 0.5 nM in RD cells (Figure 3C). Furthermore, siR-NRAS suppressed the expression levels of *NRAS* mRNA (Figure 3D). siR-NRAS mainly induced autophagy and upregulated KRAS expression (Figure 3E). In addition, we tested for changes in *NRAS*-Q61H mRNA levels in siR-NRAS or CM-MIR143#12-transfected RD cells. siR-NRAS decreased the mRNA expression of *NRAS*-Q61H at 72 h after transfection (Figure 3F). CM-MIR143#12 decreased the mRNA expression of *NRAS*-Q61H at 48 h, with a feedback-induced increase in the expression after 72 h (Figure 3G). These findings suggested that CM-MIR143#12 may strongly regulate the RAS pathway by silencing not only *KRAS* but also *NRAS*-Q61H in NRAS-mutated cells.

### 2.5. Direct Knockdown of the Pax3-Foxo1 Chimeric Gene By CM-MIR143#12 in Rh30 Cells

**Figure 4:**
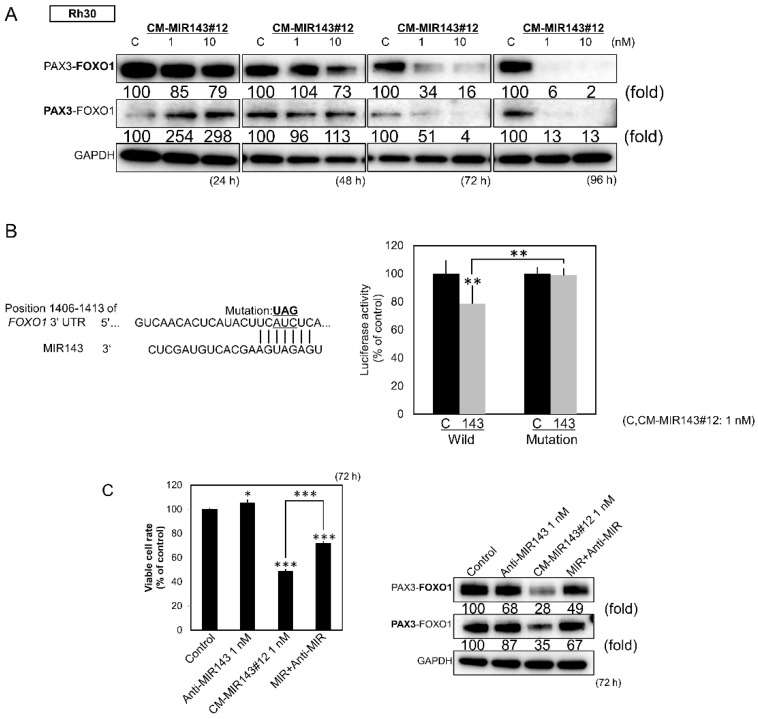
(**A**) Protein expression of PAX3–FOXO1 in Rh30 cells at 24, 48, 72, and 96 h after transfection with CM-MIR143#12 (1, 10 nM). (**B**) Luciferase activities after co-transfection with control RNA or CM-MIR143#12 and wild type or mutant pMIR vectors having the predictive MIR143 binding site in the 3′UTR of *FOXO1*. The left part of the figure shows the region of the 3′UTR of human *FOXO1* mRNA complementary to the mature MIR143. (**C**) Effect of combined treatment with antagoMIR143/CM-MIR143#12 on the growth of Rh30 cells. Cells were transfected with control RNA or CM-MIR143#12 (1 nM) + antagoMIR143 (1 nM). Left panel: cell viability; right panel: expression level of PAX3–FOXO1 assessed at 72 h after the transfection. PAX3–FOXO1 was detected using the antibody indicated in bold. Results are presented as the mean ± SD; * *p* < 0.05; ** *p* < 0.01; *** *p* < 0.001.

Next, we explored the putative target genes of MIR143-3p that would have a significant impact on growth and cell death in RMS using TargetScan. We found that the *PAX3–FOXO1* chimeric gene contained sequences with which MIR143-3p could potentially bind. Consistent with the result of in silico analysis, ectopic expression of CM-MIR143#12 significantly decreased the expression of PAX3–FOXO1 protein (Figure 4A). Moreover, the luciferase reporter assay using wild type *PAX3*–*FOXO1* 3′UTR sequences showed that CM-MIR143#12 bound to potential target sequences shown in the left part of Figure 4B, resulting in lowered luciferase activity (Figure 4B). On the other hand, the introduction of a mutation in the putative binding site markedly attenuated the inhibitory effect elicited by the ectopic expression of CM-MIR143#12 (Figure 4B). Furthermore, transfection with antagoMIR143 significantly increased the rate of cell viability and restored the expression levels of PAX3–FOXO1, which had been decreased by CM-MIR143#12 (Figure 4C).

### 2.6. Knockdown of PAX3–FOXO1 Induced Autophagy in RMS Cell Lines

**Figure 5:**
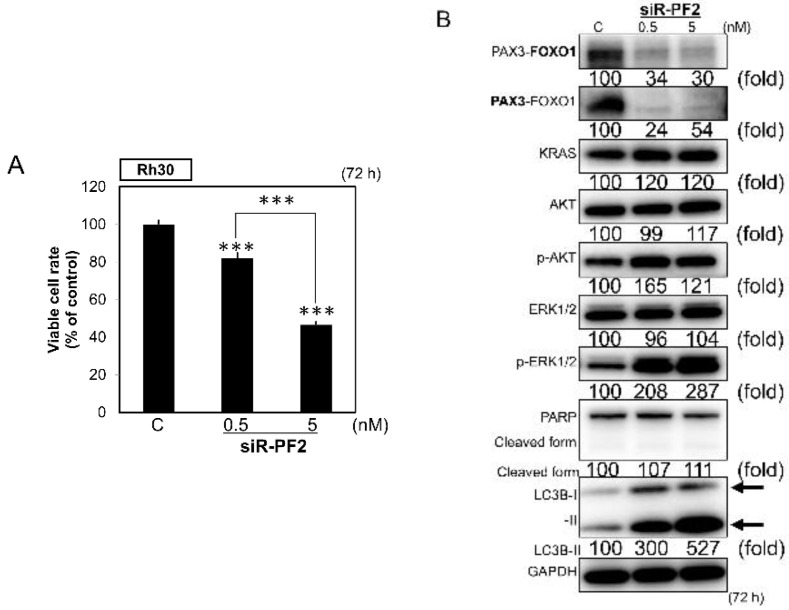
(**A**,**B**) Effects of PAX3–FOXO1 knockdown on cell growth (**A**) and protein expression levels of PAX3–FOXO1, KRAS, AKT, ERK1/2, PARP, and LC3B in Rh30 cells, as examined by Western blot analysis (**B**) 72 h after transfection of the cells with siR-PF2 at a concentration of 0.5 or 5 nM. siR-PF2 was synthesized by targeting the fusion sites between exon 7 of PAX3 and exon 2 of FOXO1. The knockdown of PAX3–FOXO1 was performed using siR-PF2 [40]. PAX3–FOXO1 was detected using the antibody indicated in bold. Results are presented as mean ± SD; *** *p* < 0.001.

To clarify the potential roles of PAX3–FOXO1 in the CM-MIR143#12-induced cell death, we evaluated the effect of *PAX3*–*FOXO1* knockdown by using siR-PF2 [40]. Transfection with this siRNA, which specifically knocked down the chimeric *PAX3*–*FOXO1* gene (siR-PF2; Figure 4B), significantly inhibited the growth of Rh30 cells (Figure 5A). siR-PF2 mainly induced autophagy with a transition from LC3B-I to LC3B-II, but without PARP cleavage (Figure 5B).

### 2.7. Tumor-Suppressing Effect of CM-MIR143#12 on Rh30 Cell-Xenografted Mice

**Figure 6:**
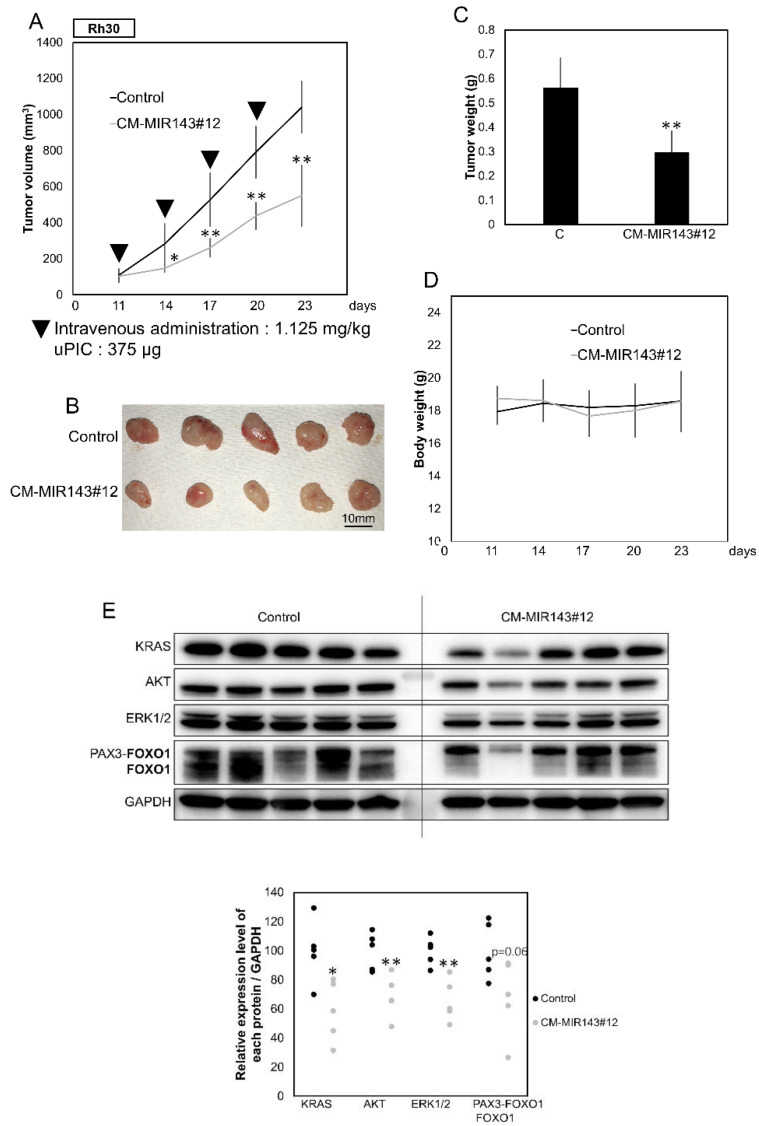
(**A**) Anti-tumor effect of CM-MIR143#12 on Rh30 cell xenografted-tumor model mice. The graph shows a time course of tumor size in mice injected with control RNA or CM-MIR143#12 (*n* = 5). Arrowheads represent intravenous injections of control RNA or CM-MIR143#12 (1.125 mg/kg/administration) delivered using polyionic copolymers (PIC; 375 µg/administration) every 3 days. (**B**) Photo of tumors in treated and control groups. (**C**) Graph showing tumor weight. (**D**) Graph showing a time course of body weight of mice injected with control RNA or CM-MIR143#12. (**E**) Panel showing Western blot detection of KRAS, AKT, ERK1/2, and PAX3–FOXO1 proteins expressed in the tumor tissues treated with control RNA or CM-MIR143#12. Relative expression levels of each protein/GAPDH were calculated based on densitometric values of each protein and β-actin. PAX3–FOXO1 was detected using the antibody indicated in bold. Results are presented as the mean ± SD; * *p* < 0.05; ** *p* < 0.01.

To further validate the tumor-suppressing effect of CM-MIR143#12, we performed an in vivo experiment in which CM-MIR143#12 was systemically administered using polyionic copolymers every 3 days (1125 μg/kg/administration), four times in total, to nude mice that had been subcutaneously inoculated with Rh30 cells (Figure S5A). As a result, significant suppression of tumor growth was observed in the group generally injected with CM-MIR143#12 compared with growth in the control group (Figure 6A–C and Figure S5B). Statistically, the effects were obvious because the *p*-values for the first and second administrations were 0.029 and 0.001, respectively (Figure 6A). There was no difference in body weight between CM-MIR143#12 and control MIRNA-treated groups (Figure 6D). Western blot analysis of the tumor samples showed results similar to those obtained in the in vitro experiment (Figure 6E). These findings indicate that CM-MIR143#12 also exerted potent tumor-suppressing activity by targeting *KRAS*, *AKT*, *ERK1/2,* and *PAX3*–*FOXO1* in vivo.

## 3. Discussion

We demonstrated that the treatment of ARMS Rh30 cells with CM-MIR143#12 resulted in significant growth suppression via interruption of the KRAS/AKT/PAX3–FOXO1 axis. In addition, CM-MIR143#12 may strongly regulate the RAS pathway by silencing not only KRAS but also NRAS-Q61H in NRAS-mutated RD cells.

MIRNA can be used to potentially target any gene that has been difficult to target using conventional molecular targeted drugs. Moreover, MIRNA can disrupt signal cascades systematically by downregulating the expression of multiple key genes involved in driving signaling cascades. The rationale for the combined treatment of MEK- and PI3K-specific inhibitors in the clinical treatment of RAS-mutated RMS has already been reported [41]. As shown in Figure 3E, siR-NRAS upregulates KRAS expression. This result suggests that silencing NRAS or KRAS alone causes a feedback mechanism to occur in a RAS-positive circuit. In light of this, MIRNAs that may be able to comprehensively inhibit pathways may be able to achieve treatments that are less likely to cause feedback mechanisms. MIR143-3p is a major MIRNA that can be directed against KRAS networks because it targets not only *KRAS* but also *AKT*, *ERK*s [26,42,43], and *SOS1* [35]. Therefore, compared with let-7 [44], MIR-217 [45], and others that target *KRAS*, MIR143-3p suppressed KRAS protein expression more strongly than the others because of the presence of two binding sites for MIR143-3p in the 3′UTR of *KRAS* mRNA. CM-MIR143#12 is superior in its ability to target MAPK/ERK1/2 and PI3K/AKT as well as upstream KRAS, NRAS, and SOS1 in RAS-mutated RMS. In the current study, tumor suppression was observed in response to an extremely low dose of CM-MIR143#12 in Rh30-xenografted mice.

In our previous study, the knockdown of *PAX3*–*FOXO1* induced autophagy by shifting expression from *PKM2* to *PKM1* by inhibiting the expression of *PTBP1*, which regulates the expression of *PKM1* and *PKM2* as a splicer in ARMS [21], thus suggesting that *PAX3*–*FOXO1* positively contributes to the maintenance of the glycolysis-dominant status. However, the protein expression of PTBP1 was almost unchanged in Rh30 cells transfected with CM-MIR143#12. Thus, another mechanism could exist in the case of CM-MIR143#12-induced autophagy. Of note, this is what distinguishes this result from those of previous reports. MIR143 has been reported to inhibit autophagy [46,47] and the induction of autophagy by CM-MIR143#12 may be one of the processes leading to apoptosis; this is an issue that needs to be clarified in future studies.

CM-MIR143#12 shows anticancer effects by impairing the KRAS signaling networks in many kinds of cancer [35,36,37,48]. Notably, in the current study, CM-MIR143#12 showed anticancer effects on RMS by targeting the ARMS-specific chimeric gene *PAX3*–*FOXO1*, which plays an important role in promoting cell cycle progression in ARMS [40]. Moreover, CM-MIR143#12 downregulated the expression of FGFR4, that is, the downstream of PAX3–FOXO1 (data not shown). These results suggest that the RAS pathway can be more strongly controlled by CM-MIR143#12. Recently, MIRNA delivery via LNP successfully suppressed xenografted tumors [49]. In the current study, we also efficiently inhibited tumor growth by using uPIC, the diameter of which is approximately 20 nm and which was selectively prepared as a charge-matched polyionic complex (Figure S4A) [36,37,50].

Treatment of RMS consists of a combination of chemotherapy and radiotherapy after tumor resection or biopsy. The current main therapy for RMS involves a combination of vincristine, actinomycin D, and cyclophosphamide (VAC). In a recent clinical trial [12,13], molecular targeted drugs were tested. The prognosis was improved in the low-risk group with fusion-negative RMS. Although convalescence was improved, the results were still poor for the *PAX3*–*FOXO1*-positive RMS group. Therefore, MIRNA or siRNA are potential new therapeutic drugs that can be directed toward *PAX3*–*FOXO1*-positive RMS. RNA medicine against RMS will hopefully be successful when introduced via drug delivery systems (DDS) such as lipid nanoparticles (LNP; [51]) and polymers [52] because blood vessels are abundant in tumor tissues, as we found in the case of the xenograft tumors of Rh30 cells (data not shown). Moreover, CM-MIR143#12, which is RNase-resistant (Figure S1B), remains undegraded in the blood for several hours. There may be potential for CM-MIR143#12 delivery due to the enhancement of blood retention.

## 4. Materials and Methods

### 4.1. Cell Culture and Cell Viability

Human ERMS (RD and RMS-YM) and ARMS (SJ-Rh30 [Rh30] and SJ-Rh41 [Rh41]) cell lines were used. The RD cell line was obtained from the Japanese Collection of Research Bioresources (JCRB) Cell Bank. RMS-YM cell lines were obtained from RIKEN CELL BANK (Tsukuba, Japan). ARMS cell lines were provided by Dr. H. Hosoi (Kyoto Prefectural University of Medicine). Eagle’s minimal essential medium was used to culture the human ERMS cell line RD while RPMI-1640 medium was used to culture the human ERMS cell line RMS-YM and the human ARMS cell lines Rh30 and Rh41. All media were supplemented with 10% (*v/v*) heat-inactivated FBS (Sigma-Aldrich Co, St. Louis, MO, USA) and 2 mM L-glutamine, and the cells were incubated under an atmosphere of 95% air and 5% CO_2_ at 37 °C. Cell line authentication was conducted using short tandem repeat (STR) analysis, and the cells were tested for mycoplasma contamination. STR analysis was performed by using primers of TH01, TPOX, vWA, Amelogenin, CSF1PO, D16S539, D7S820, D13S317, D5S818, and D21S11 (GenePrint^®^ 10 System; Promega, Madison WI, USA). Testing for mycoplasma contamination was conducted by exploiting the activity of certain mycoplasmal enzymes (MycoAlert™ mycoplasma detection kit; Lonza, Bazel, CHE). The number of viable cells was determined using trypan blue dye exclusion assay.

### 4.2. Transfection Experiments

All cells were seeded in 6-well plates at a concentration of 0.5 × 10^5^ per well (10–30% confluence) one prior to transfection. The mature CM-MIR143#12, antagoMIR143-3p (mirVanaTM MIRNA inhibitor; Ambion) or siRNAs for KRAS, NRAS, PAX3–FOXO1 (siR-KRAS, siR-NRAS, and siR-PF2, respectively; Invitrogen, Carlsbad, CA), and AKT (siR-AKT; CST) were used for the transfection of the cells, which was achieved using cationic liposomes, Lipofectamine^TM^ RNAiMAX (Invitrogen) according to the manufacturer’s lipofection protocol. The nonspecific control RNA (HSS, Hokkaido, Japan) sequence was 5′-GUAGGAGUAGUGAAAGGCC-3′, which was used as a control for nonspecific effects. The sequence of the CM-MIR143#12 used in this study was from a previous publication ([35]; Figure S1A). The sequence of siR-KRAS was 5′-AAUGCAUGACAACACUGGAUGACCG-3′, siR-NRAS was 5′-UACAGAGUAGCACUGCUCCAGAAGG-3′, and siR-PF2 was 5′-UGAAUUCUGAGGUGAGAGG-3′. The effects manifested by the introduction of CM-MIR143#12, and siRNAs into the cells were assessed at 24, 48, 72, or 96 h after transfection.

### 4.3. Inhibitor Agents

AKT inhibitor IV (Calbiochem, San Diego, CA, USA) without a drug delivery system was used for treating cells. The effects manifested in the cells by the AKT inhibitor were assessed at 72 h.

### 4.4. Western Blotting

Whole cells were homogenized in chilled lysis buffer comprising 10 mM Tris-HCl (pH 7.4), 1% NP-40, 0.1% deoxycholic acid, 0.1% SDS, 150 mM NaCl, 1 mM EDTA, 2% protease inhibitor cocktail, and 2% phosphatase inhibitor cocktail 2 and 3 (Sigma-Aldrich Co., St. Louis, MO, USA) and incubated for 20 min on ice. After centrifugation at 13,000 rpm for 20 min at 4 °C, the supernatants were collected as whole-cell protein samples. Protein contents were measured using a DC Protein assay kit (BIORAD, Hercules, CA, USA). Lysate protein (10 μg) was separated by SDS-PAGE using 10.0% or 12.5% polyacrylamide gels and electroblotted onto Immobilon^®^-P Transfer PVDF membranes (Millipore, Billerica, MA, USA). After blockage of nonspecific binding sites for 1 h with 20% PVDF Blocking Reagent for Can Get Signal^®^ (TOYOBO Co., LTD., Osaka, Japan) in TBS containing 0.1% Tween 20 (TBS-T), the membrane was incubated overnight at 4 °C with primary antibodies. The next day, the membrane was then washed three times with TBS-T, incubated further with secondary antibodies at room temperature for 1 h, and then washed three times with TBS-T. The immunoblots were visualized by the use of Immobilon^®^ Forte Western HRP Substrate (MILLIPORE). The primary antibodies used are as follows: anti-AKT, phospho-AKT (Ser473), ERK1/2, phospho-ERK1/2, PARP, LC3B, GAPDH, FOXO1 (Cell Signaling Technology [CST], Inc., Danvers, MA, USA); anti-KRAS (LifeSpan BioScience, Seattle, WA, USA); and anti-PAX3 (R&D systems, Minneapolis, MN, USA). HRP-conjugated goat anti-rabbit and horse anti-mouse IgG (CST) were used as secondary antibodies. GAPDH was used as an internal control. Immunoblot images were acquired by using an ImageQuant™ LAS4000 biomolecular imager (GE Healthcare Life Sciences; Pittsburgh, PA, USA). Original unedited blots can be found at Figure S6.

### 4.5. Quantitative Real-Time PCR (qPCR)

Total RNA was isolated from cultured cells or tumor tissues using a NucleoSpin miRNA isolation kit (TaKaRa, Otsu, Japan). RNA concentrations and purity were assessed by UV spectrophotometry. RNA integrity was checked by formaldehyde gel electrophoresis. To determine the expression levels of MIR143-3p, we conducted quantitative real-time PCR (qPCR) using TaqMan MicroRNA Assays (Applied Biosystems) and THUNDERBIRD^TM^ Probe qPCR Mix (TOYOBO) according to the manufacturer’s protocol. RNU6B was used as an internal control. For determination of the expression levels of NRAS and glyceraldehyde-3-phosphate dehydrogenase (GAPDH) mRNAs, total RNA was reverse-transcribed with a PrimeScript RT reagent Kit (TaKaRa). qPCR was then performed with specific primers for each gene using THUNDERBIRD^TM^ Next SYBR qPCR Mix (TOYOBO). The primers for NRAS and GAPDH were as follows: NRAS-sense—5′-CCT CCT CAC TTG GCT GTC TG-3′—and NRAS-antisense—5′-TCA CGT TTG CGG TTT GGT TC-3′; NRAS-common sense—5′-CCC GGC TGT GGT CCT AAA TC-3′—and NRAS wild type-antisense—5′-TCA TGG CAC TGT ACT CTT CT-3′—or NRAS Q61H-antisense—5′- TCA TGG CAC TGT ACT CTT CA-3′; GAPDH-sense—5′-TCT AGA CGG CAG GTC AGG TCC ACC-3′ and GAPDH-antisense—5′-CCA CCC ATG GCA AAT TCC ATG GCA-3′. RNU6B and GAPDH were used as internal controls. The relative expression levels were calculated using the ΔΔCt method.

### 4.6. Luciferase Reporter Assay

Searching the TargetScan database (TargetScan Release 7.2 [53]) to find algorithm-based binding sites of MIR143-3p, we found the predicted binding site to be at position 1406–1413 for MIR143-3p in the 3′UTR of *FOXO1* mRNA. The sequence region containing the putative binding sequence of MIR143-3p was inserted into a pMIR-REPORT™ Luciferase MIRNA Expression Reporter Vector (Applied Biosystems) according to the manufacturer’s protocol. Moreover, we made other pMIR constructs, including one encompassing a mutated seed sequence for MIR143-3p (wild type, UCAUCUCA; mutant, UCUAGUCA) using a PrimeSTAR^®^ Mutagenesis Basal Kit (TaKaRa). The mutation of the vector was confirmed by sequence analysis. pRL-TK Renilla Luciferase Reporter vector (Promega) was used as an internal control vector. Rh30 cells were seeded into 96-well plates at a concentration of 0.5 × 10^4^ per well on the day before the transfection. The cells were co-transfected with either reporter vector (0.01 μg/well each) and 1 nM CM-MIR143#12 or nonspecific non-coding siRNA (Dharmacon, Tokyo, Japan). Luciferase activities were measured at 24 h after co-transfection by using a Dual-Glo Luciferase Assay System (Promega) according to the manufacturer’s protocol. Luciferase activities were reported as the firefly luciferase/Renilla luciferase ratio.

### 4.7. Hoechst 33342 Staining

Hoechst 33342 (5 μg/mL; Sigma-Aldrich) of transfected Rh30 cells was performed 72 h after transfection. The cells were then incubated at 37 °C for 1 h and thereafter washed with PBS. After the wash, cells were resuspended and examined with an Olympus microscope (Tokyo, Japan) equipped with an epi-illuminator and appropriate filters. The apoptotic cells were characterized by the presence of condensed and/or fragmented nuclei.

### 4.8. Electron Microscopy

Rh30 cells treated with control RNA or CM-MIR143#12 (1 nM) were harvested and rinsed with PBS. The cells were then fixed for 2 h with 2% paraformaldehyde and 2.5% glutaraldehyde in 0.2 M phosphate buffer (pH 7.4, PB), rinsed in PB, and postfixed in 2% osmium tetraoxide for 2 h. After having been washed with PB, the cells were progressively dehydrated by passage through a 10% graded series of 30–100% ethanol and then cleared in QY-1 (Nissin EM, Tokyo, Japan). Thereafter, they were embedded in Epon 812 resin (TAAB Laboratories Equipment, Reading, Aldermaston, Berks, Berks, RG7 8NA, UK), after which thin sections (70 nm thickness) were prepared. Finally, the sections were stained with uranyl acetate and lead citrate and examined by transmission electron microscopy using a Hitachi-7650 (Hitachi, Tokyo, Japan), operating at 80 kV.

### 4.9. Immunofluorescence (IFC) Study

Cells of each cell type were seeded into the wells of a Lab-Tek II Chamber Slide System (Thermo Fisher Scientific Inc., Waltham, MA, USA). After transfection with a given MIRNA, including control RNA, the cells were immunostained with an anti-LC3B antibody according to the immunofluorescence protocol of CST. Next, the nuclei were stained with Hoechst 33342, and for actin labeling, the cells were incubated with a fluorescent F-actin probe, rhodamine phalloidin (Cytoskeleton Inc., Denver, CO, USA). Finally, the cells were observed with a BIOREVO fluorescence microscope (Keyence, Osaka, Japan).

### 4.10. Human Tumor Xenograft Model

Animal experimental protocols were approved by the Committee for Animal Research and Welfare of Gifu University (project identification code: H30-42, 10 April 2018). BALB/cSLC-nu/nu (nude) mice were obtained from Japan SLC (Hamamatsu, Japan). Human rhabdomyosarcoma Rh30 cells were inoculated at 2.0 × 10^6^ cells/100 μL per site into the back of each of several mice. The inoculation day was set as Day 0. At 11 days after inoculation, we confirmed the engraftment of the tumors. For administration to the mice, MIRNA was mixed with poly(ethylene glycol)-poly (Ornithine) copolymer (a gift from Professor Kazunori Kataoka of the University of Tokyo) to obtain MIRNA-loaded polyionic copolymers (PIC; unit PIC, uPIC) [50] for delivery of the MIRNA to the tumors. After control RNA or CM-MIR143#12 (1.125 mg/kg per single administration) in 120 μL of 10 mM HEPES had been incubated with 15 μL of PIC, the mixture was injected into the tumor every 3 days. Each group contained 5 mice. In the cases in which the tumors had disappeared, we stopped the injections. The tumor volume was calculated using the following formula: 0.5236 L1 (L2)^2^, where L1 is the long axis and L2 is the short axis of the tumor. This formula is described in a previous report [22,54].

### 4.11. RNA Samples from Skeletal Muscle Cells

Skeletal muscle cell total RNA (ScienCell Research Laboratories) was used for qPCR as a noncancerous control.

### 4.12. Statistics

Each examination was performed in triplicate. For in vitro and in vivo experiments, statistical significance of differences was evaluated by performing the two-sided Student’s t-test. The values were presented as the mean ± standard deviation. A *p*-value < 0.05 was considered to indicate statistically significant differences.

## 5. Conclusions

**Figure 7:**
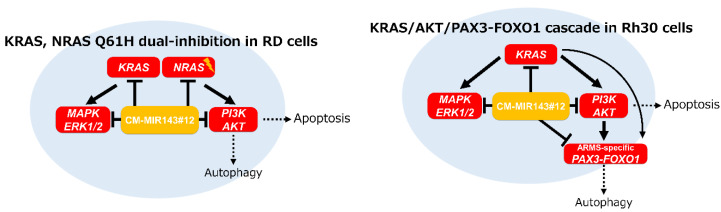
Schematic diagram showing the mechanism of growth inhibition by the ectopic expression of CM-MIR143#12 in RD and Rh30 cells.

In conclusion, this study demonstrates targeting of KRAS/NRAS Q61H in RD cells and the KRAS/PAX3–FOXO1 cascade in Rh30 cells using CM-MIR143#12; CM-MIR143#12 could efficiently impair these pathways by silencing key genes in the KRAS/NRAS Q61H/AKT network in RD cells or KRAS/AKT/PAX3–FOXO1 network in Rh30 cells (Figure 7). We demonstrated the possibility that CM-MIR143#12 can be a new nucleic acid medicine for the treatment of RMS.

## Supplementary Materials

The following are available online at https://www.mdpi.com/2072-6694/12/11/3312/s1. Figure S1: CM-MIR143#12 details. Figure S2: Effects of ectopic expression of CM-MIR143#12 on RMS and normal fibroblast cells. Figure S3: Effects of ectopic expression of siR-AKT on RD and Rh30 cells. Figure S4: The mRNA expression of wild type or mutation type of NRAS in RD and Rh30 cells. Figure S5: Diagram showing the formulation of uPIC with CM-MIR143#12 and changing in the tumor size of each mouse. Figure S6: Original unedited blots from primary figures.
