# Supplementary material for: Synthetic MIR143-3p Suppresses Cell Growth in Rhabdomyosarcoma Cells by Interrupting RAS Pathways Including PAX3–FOXO1
Source: Cancers (Basel). 2020 Nov 10;12(11):3312. doi: 10.3390/cancers12113312 (PMC7696565; doi:10.3390/cancers12113312)
Supplement: Supplementary file 1 [file cancers-12-03312-s001.pdf]

Supplementary Materials:

Synthetic MIR143-3p Suppresses Cell Growth in Rhabdomyosarcoma Cells by Interrupting RAS Pathways Including PAX3–FOXO1

Nobuhiko Sugito, Kazuki Heishima, Yuko Ito and Yukihiro Akao

A CM-MIR143

No	Type	Sequence
#1	Wild	S: 3′-GGUCUCUACGUCGUGACGUGGAGU-5′<br>AS: 5′-UGAGAUGAAGCACUGUAGCUCAGG-3′
#12	F/Ome	S: 3′-GGU C UCUACGUCGUGACGUGGAGU-5′<br>AS: 5′-U^G^AGAUGAAGCACUGUAGCUCA^dT^dT-3′

N: 2’-F RNA
N: 2’-Ome RNA
N: RMA
^: PS
_: mismatch

B

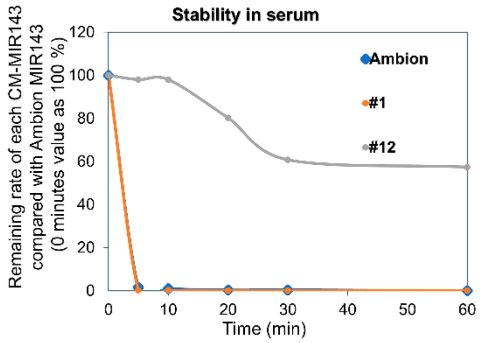

Figure S1. CM-MIR143#12 details. (**A**) RNA sequences of CM-MIR143. CM-MIR143#1 is wild type of MIR143. CM-MIR143#12 is F/Ome-modified MIR143. F RNA, Fluoro-RNA; Ome RNA, O-Methyl RNA; PS, phosphorothioate. (**B**) Remaining percentage of each MIR143, Ambion (Applied Biosystems, Foster City, CA, USA), #1, and #12 remaining in the presence of FBS evaluated by performing RT-qPCR. The 0-min value of each MIR143 is indicated as 100%. The mean value was taken for each time.

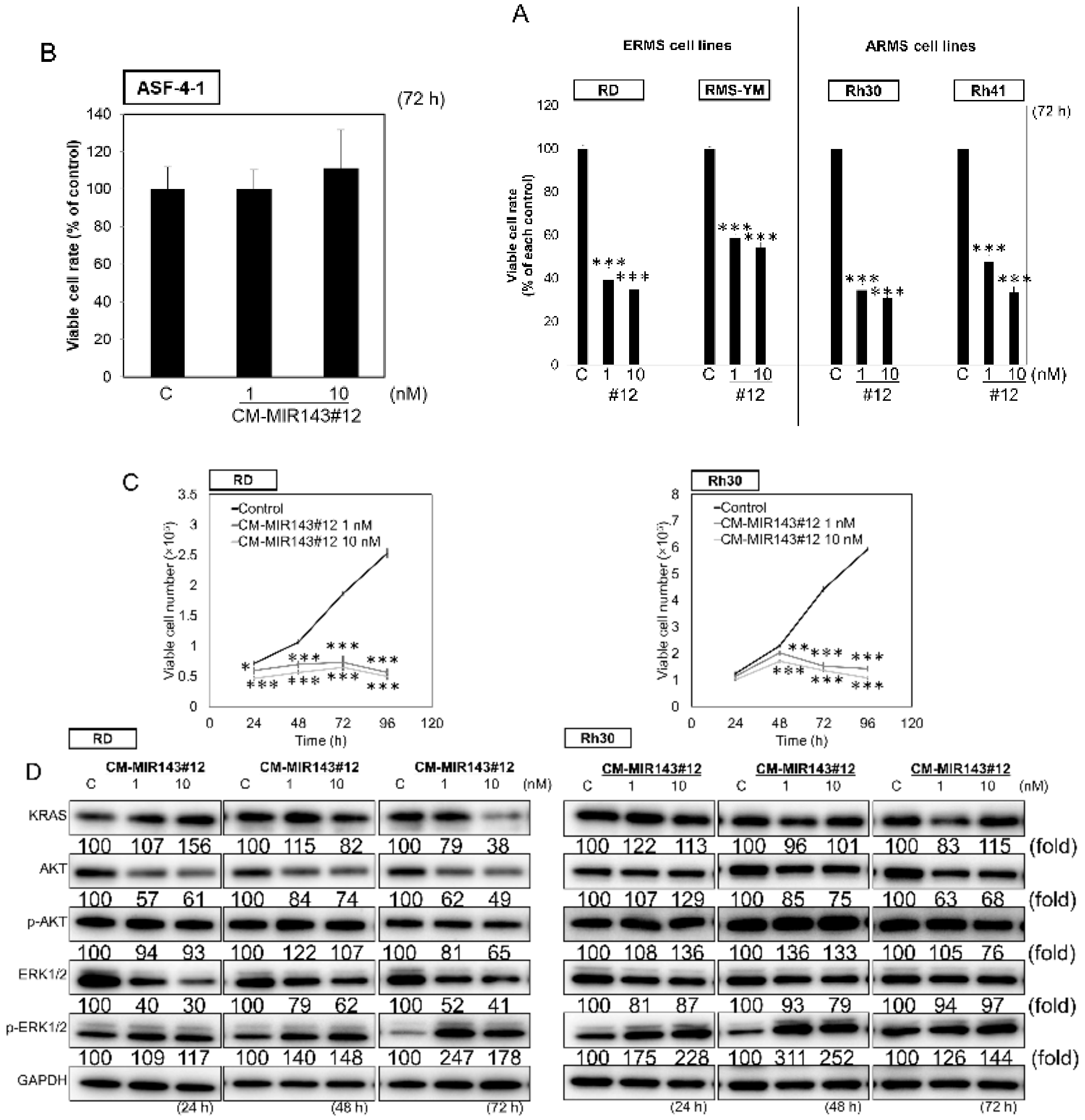

Figure S2. Effects of ectopic expression of CM-MIR143#12 on RMS and normal fibroblast cells. (**A**,**B**) Effects of ectopic expression of CM-MIR143#12 on viability of all RMS (**A**) and normal fibroblast (**B**) cells at 72 h. (**C**, **D**) Effects of ectopic expression of CM-MIR143#12 on cell viability of RMS cells (**C**) and expression of KRAS, AKT, ERK1/2 (**D**), and PARP as an apoptotic marker and LC3B as an autophagic one estimated by Western blot analysis (**D**) at 24, 48, 72, and 96 h after transfection of RD and Rh30 cells with CM-MIR143#12 at a concentration of 1 or 10 nM. Results are presented as the mean ± SD; * $p < 0.05$; ** $p < 0.01$; *** $p < 0.001$.

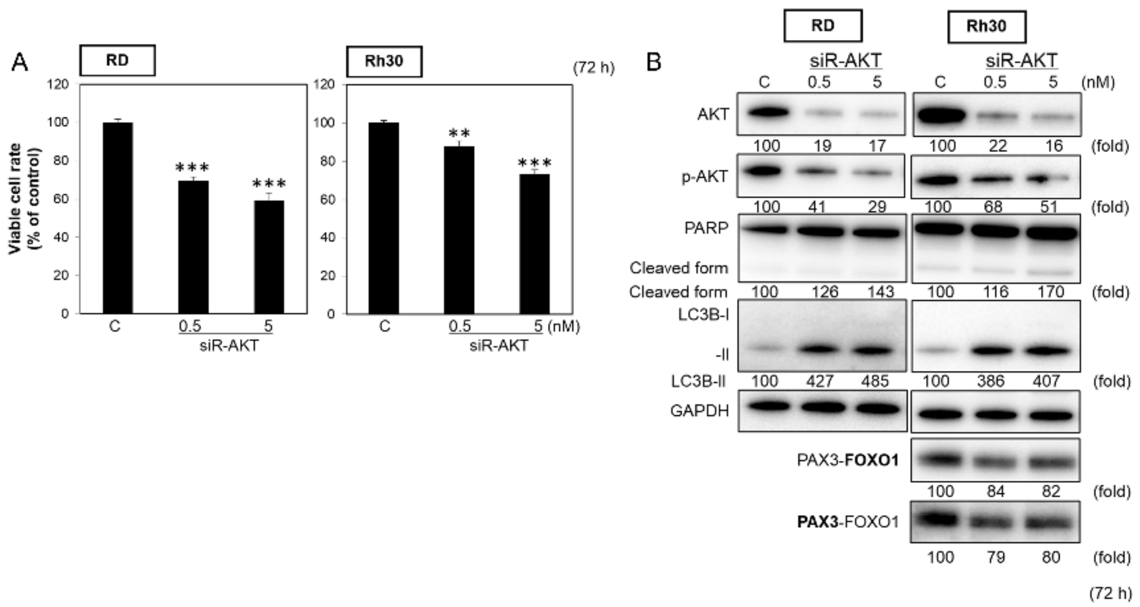

Figure S3. Effects of ectopic expression of siR-AKT on RD and Rh30 cells. (A, B) Effects of siR-AKT on cell viability of RMS cells (**A**) and expression of AKT, p-AKT, PARP, LC3B, and PAX3-FOXO1 estimated by Western blot analysis (**B**) at 72 h after treatment of RD and Rh30 cells with siR-AKT at a concentration of 0.5 or 5 nM. PAX3-FOXO1 was detected using the antibody indicated in bold. Results are presented as the mean ± SD; ** $p < 0.01$; *** $p < 0.001$.

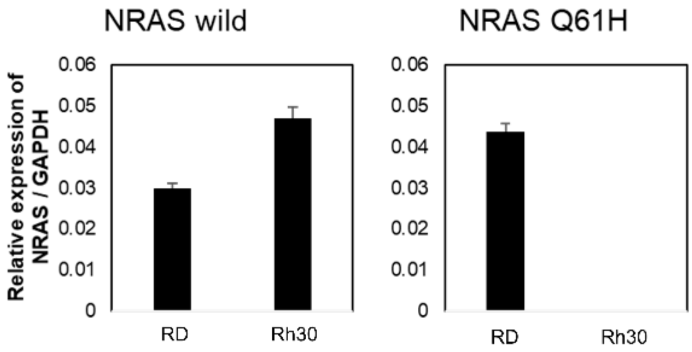

Figure S4. The mRNA expression of wild type or mutation type of *NRAS* in RD and Rh30 cells.

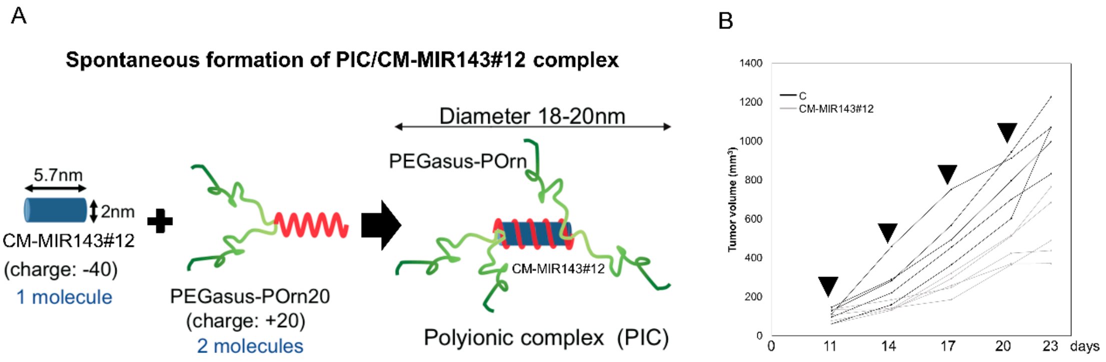

Figure S5. Diagram showing the formulation of uPIC with CM-MIR143#12 (**A**) and changing in the tumor size of each mouse (**B**).

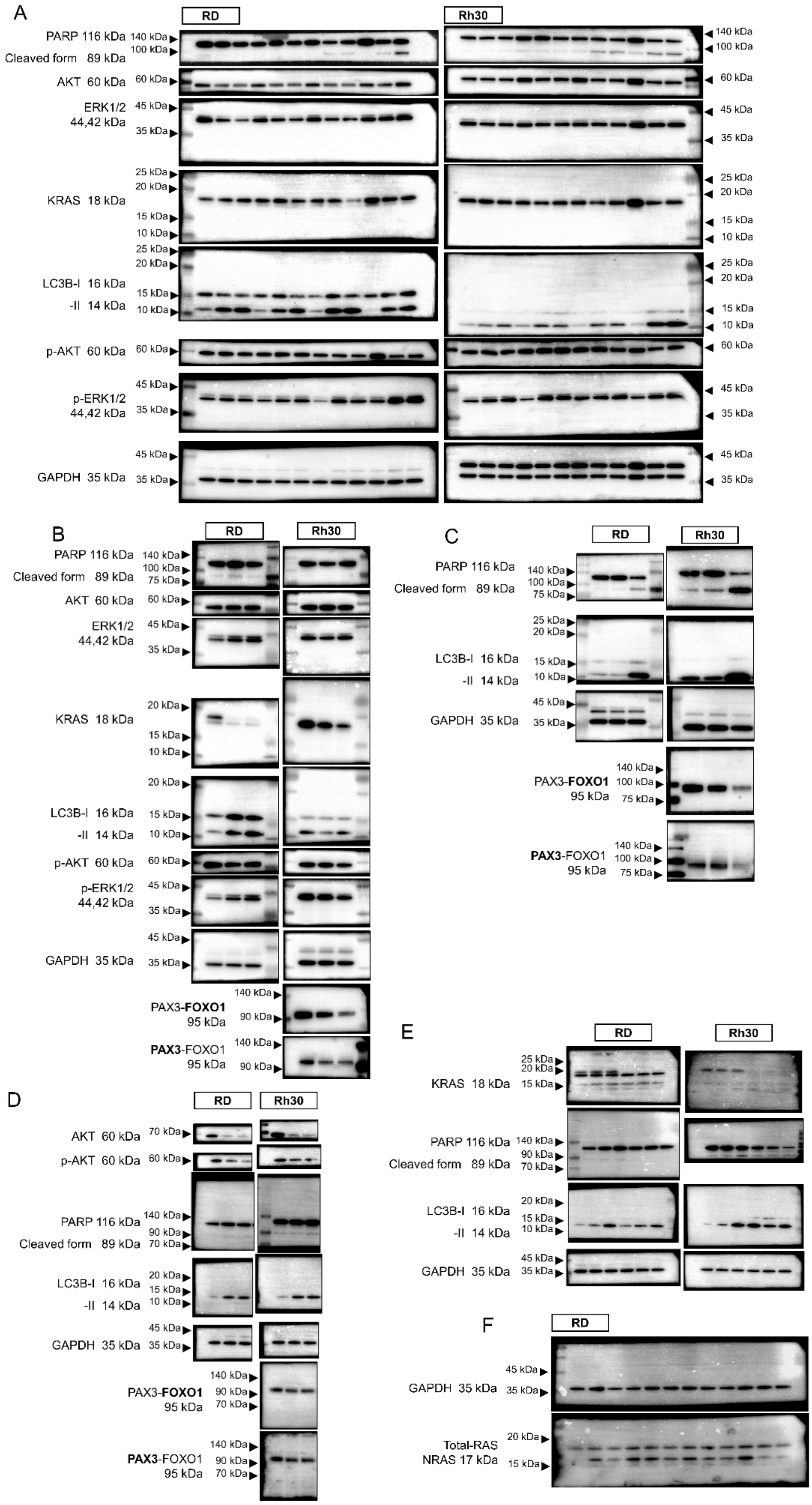
A
RD
Rh30
PARP 116 kDa
Cleaved form 89 kDa
AKT 60 kDa
ERK1/2 44,42 kDa
KRAS 18 kDa
LC3B-I 16 kDa
-II 14 kDa
p-AKT 60 kDa
p-ERK1/2 44,42 kDa
GAPDH 35 kDa
140 kDa
100 kDa
60 kDa
45 kDa
35 kDa
25 kDa
20 kDa
15 kDa
10 kDa
B
RD
Rh30
PARP 116 kDa
Cleaved form 89 kDa
AKT 60 kDa
ERK1/2 44,42 kDa
KRAS 18 kDa
LC3B-I 16 kDa
-II 14 kDa
p-AKT 60 kDa
p-ERK1/2 44,42 kDa
GAPDH 35 kDa
PAX3-**FOXO1** 95 kDa
PAX3-FOXO1 95 kDa
140 kDa
100 kDa
90 kDa
75 kDa
60 kDa
45 kDa
35 kDa
20 kDa
15 kDa
10 kDa
C
RD
Rh30
PARP 116 kDa
Cleaved form 89 kDa
LC3B-I 16 kDa
-II 14 kDa
GAPDH 35 kDa
PAX3-**FOXO1** 95 kDa
PAX3-FOXO1 95 kDa
140 kDa
100 kDa
75 kDa
45 kDa
35 kDa
25 kDa
20 kDa
15 kDa
10 kDa
D
RD
Rh30
AKT 60 kDa
p-AKT 60 kDa
PARP 116 kDa
Cleaved form 89 kDa
LC3B-I 16 kDa
-II 14 kDa
GAPDH 35 kDa
PAX3-**FOXO1** 95 kDa
PAX3-FOXO1 95 kDa
140 kDa
90 kDa
70 kDa
60 kDa
45 kDa
35 kDa
20 kDa
15 kDa
10 kDa
E
RD
Rh30
KRAS 18 kDa
PARP 116 kDa
Cleaved form 89 kDa
LC3B-I 16 kDa
-II 14 kDa
GAPDH 35 kDa
140 kDa
90 kDa
70 kDa
45 kDa
35 kDa
25 kDa
20 kDa
15 kDa
10 kDa
F
RD
GAPDH 35 kDa
Total-RAS
NRAS 17 kDa
45 kDa
35 kDa
20 kDa
15 kDa

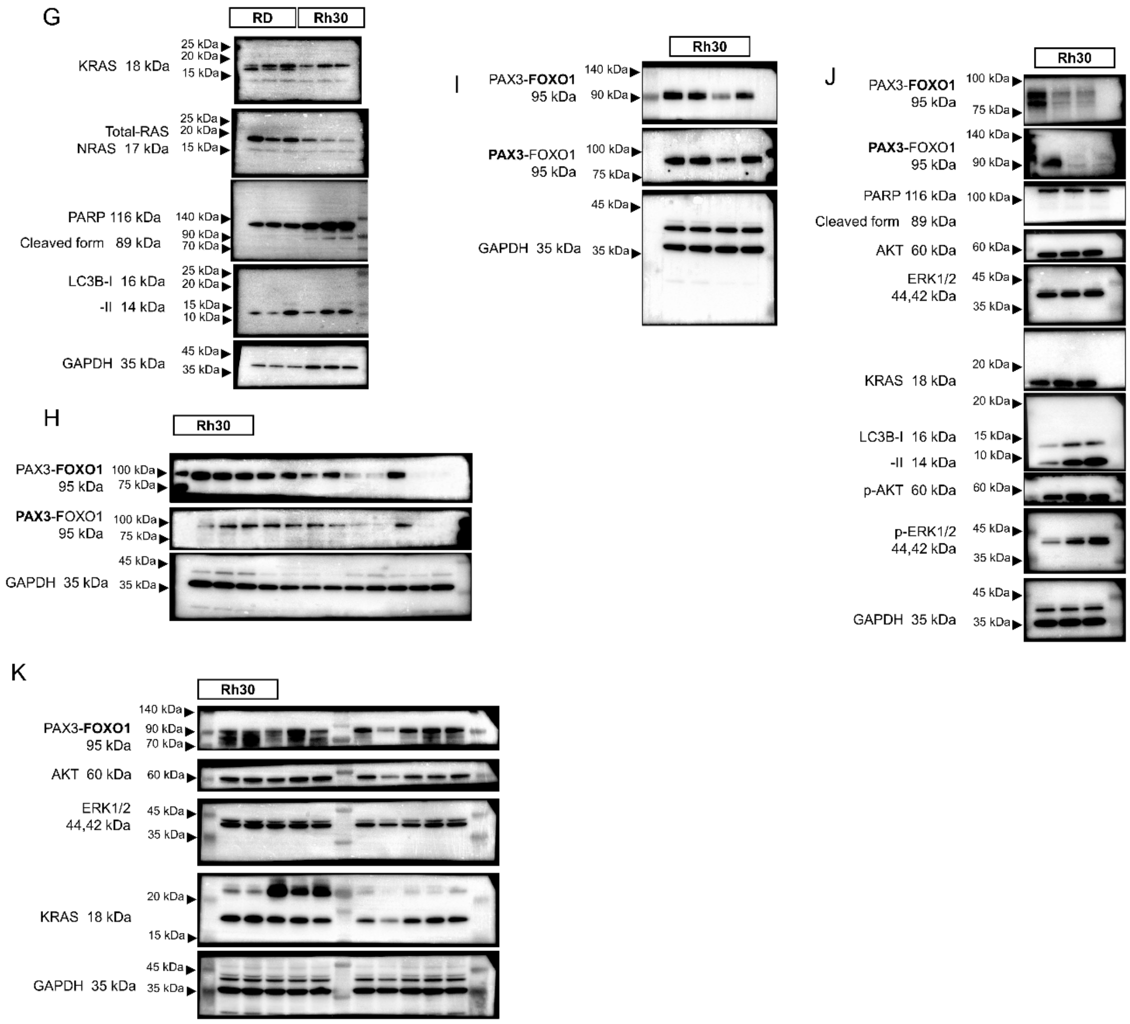

Figure S6. Original unedited blots from primary figures. (**A**) Figure S2D and Figure 1D, (**B**) Figure 2B, (**C**) Figure 2D, (**D**) Figure S3, (**E**) Figure 2F, (**F**) Figure 3B, (**G**) Figure 3E, (**H**) Figure 4A, (**I**) Figure 4C, (**J**) Figure 5B, (**K**) Figure 6E.

© 2020 by the authors. Licensee MDPI, Basel, Switzerland. This article is an open access article distributed under the terms and conditions of the Creative Commons Attribution (CC BY) license (http://creativecommons.org/licenses/by/4.0/).
